# A Cost-Utility Analysis of Robotic Arm-Assisted Total Hip Arthroplasty: Using Robotic Data from the Private Sector and Manual Data from the National Health Service

**DOI:** 10.1155/2022/5962260

**Published:** 2022-02-27

**Authors:** N. D. Clement, P. Gaston, D. F. Hamilton, A. Bell, P. Simpson, G. J. Macpherson, J. T. Patton

**Affiliations:** ^1^Edinburgh Orthopaedics, The Royal Infirmary of Edinburgh, Little France, Edinburgh EH16 4SA, SCT, UK; ^2^Department of Orthopaedics, School of Clinical Sciences, University of Edinburgh, Edinburgh, SCT, UK; ^3^Spire Murrayfield Hospital, 122 Corstorphine Road, Edinburgh EH12 6UD, SCT, UK; ^4^School of Health and Social Care, Edinburgh Napier University, Edinburgh, SCT, UK

## Abstract

**Purpose:**

The aim was to assess the cost-effectiveness of robotic arm-assisted total hip arthroplasty (rTHA) compared with manual total hip arthroplasty (mTHA) and to assess the influence of annual volume on the relative cost-effectiveness of rTHA.

**Methods:**

A database of both rTHA (*n* = 48 performed in a private centre) and mTHA (*n* = 512 performed in the National Health Service) was used. Patient demographics, preoperative Oxford hip score, forgotten joint score, EuroQol 5-dimensional 3-level (EQ-5D), and postoperative EQ-5D were recorded. Two models for incremental cost-effectiveness ratios using cost per quality-adjusted life year (QALY) for rTHA were calculated based on a unit performing 100 rTHAs per year: 10-year follow-up and a lifetime time horizon (remaining life expectancy of a 69-year-old patient).

**Results:**

When adjusting for confounding factors, rTHA was independently associated with a 0.091 (*p*=0.029) greater improvement in the EQ-5D compared to mTHA. This resulted in a 10-year time horizon cost per QALY for rTHA of £1,910 relative to mTHA, which increased to £2,349 per QALY when discounted (5%/year). When using the 10-year time horizon cost per QALY was approximately £3,000 for a centre undertaking 50 rTHAs per year and decreased to £1,000 for centre undertaking 200 rTHAs per year. Using a lifetime horizon, the incremental unadjusted cost per QALY gained was £980 and £1432 when discounted (5%/year) for rTHA compared with mTHA.

**Conclusions:**

Despite the increased cost associated with rTHA, it was a cost-effective intervention relative to mTHA due to the associated greater health-related quality of health gain, according to the EQ-5D outcome measure.

## 1. Introduction

Despite total hip arthroplasty (THA) being declared the operation of the last century, offering good functional outcome and high satisfaction rates, robotic arm-assisted surgery has the potential to enhance the outcome further. [1, 2] The MAKO Robotic Arm Interactive Orthopaedic (RIO) system (Stryker; Kalamazoo, MI, USA) is a semi-active system (surgeon required) and was first used to perform rTHA in 2010 with subsequent FDA approval in 2015. [2] There is a growing body of evidence that demonstrates that rTHA improves component positioning accuracy when compared to manual (m)THA [3] and has been demonstrated to offer greater functional benefit over mTHA [4, 5]. Whether this improvement in functional outcome is cost-effective is not clear [3, 6].

THA has been shown to be one of the most cost-effective interventions in medicine [7], and more than 100,000 are performed annually in the UK. [8] One method of assessing cost-effectiveness is to perform a cost-utility analysis to calculate the cost of quality-of-life years (QALYs) gained by the intervention. [9, 10] The cost-effectiveness of rTHA in the UK healthcare setting is not known. Maldonado et al. [6] conducted a Markov study (using published utilities, mortality, and revisions rates) using American healthcare costs and demonstrated rTHA to be more cost-effective than mTHA. However, they did not account for the increased costs of the robot or the costs associated with preoperative imaging and intraoperative consumables, which are required relative to mTHA. Furthermore, it is not clear whether rTHA is associated with an increased health-related quality of life compared with mTHA with only Domb et al. [11] demonstrating a significant improvement in physical health using the 12-Item Short Form (SF)-12 score in contrast to Bukowski et al. [12].

QALY is a generic measure of a person's or group's state that reflects their quality of life, which is dependent on time lived in that state. One QALY is equivalent to 1 year of life lived in perfect health. To generate a QALY, health utilities are required with preference weights that equate to value or desirability and are measured on a scale of zero to one, where zero represents death and one indicates full health. The cost per QALY can then be assessed according to the cost of the intervention: cost of intervention/QALYs gained. This incremental cost-effectiveness ratio can then be calculated, and this is a widely employed methodology to assess the cost-effectiveness of an intervention and is termed a cost-utility analysis [7].

The primary aim of this study was to assess the cost-effectiveness of rTHA compared with mTHA for the management of end-stage arthritis of the hip 10 years following surgery and for an average patient's lifetime. The secondary aim was to assess the influence of number of rTHA procedures a unit undertakes per year on the relative cost-effectiveness of rTHA [3].

## 2. Patients and Methods

Ethical approval was obtained from the regional ethics committee (Research Ethics Committee, Southeast Scotland Research Ethics Service, Scotland, 11/AL/0079) for collection, analysis, and publication of the anonymised data for the mTHA cohort. Approval from the hospital was also obtained for the use of the data for the rTHA cohort as part of ongoing assessment of a new surgical process.

### 2.1. Patients

Patients were recruited from two centres and underwent THA by the same surgeons, one centre offered rTHA (private healthcare service) and the other centre only performed mTHA (National Healthcare Service (NHS)). Inclusion criteria included the following: osteoarthritis of the hip (complete radiographic joint space loss). Exclusion criteria included the following: inflammatory arthritis, haemochromatosis, chondrocalcinosis, or haemophilia, immobility, or other neurological conditions affecting musculoskeletal function. A consecutive series of patients undergoing rTHA from one centre over a 22-month period had prospective data collection of which 48 patients had 6- to 12-month postoperative data available. At the other centre, 512 patients underwent a mTHA for osteoarthritis over a 12-month period.

### 2.2. Preoperative Planning

All surgeons (PG, PS, GJM, and JTP) performed preoperative templating on all patients using standing plain anteroposterior pelvic radiographs using digital software (KODAK^©^ picture archiving and communication system on a liquid crystal display). Patients undergoing rTHA also had preoperative CT scans of the pelvis and proximal femur to aid implant positioning using patient-specific computer-aided design models using the MAKOplasty total hip application system (Stryker, Kalamazoo). The aid of templating was to restore the native centre of rotation and offset using the contralateral side as reference, if normal, and to correct for any leg length discrepancy. Planned acetabular cup position was 40 degrees of inclination and 20 to 30 degrees of anteversion in both groups.

### 2.3. Surgical Technique

During the study period, four of the authors (PG, PS, GJM, and JTP) performed all the THAs at both centres. A posterior approach to the hip joint was utilised in all patients. For those undergoing rTHA, registration pins were placed in the pelvis onto which the arrays were mounted, and computer registration was performed by mapping of prespecified anatomical landmarks. Patients undergoing rTHA received an uncemented Trident Tritanium Solid Acetabular Shell (Stryker, Kalamazoo) with a highly crosslinked polyethylene liner. Patients undergoing mTHA received a cemented crosslinked contemporary acetabular component (Stryker, Newbury, UK). All patients received a cemented Exeter stem (Stryker, Newbury, UK), which was inserted using fourth-generation cementing techniques. All patients received systemic prophylactic antibiotics (1.5 g cefuroxime before surgery and 750 mg eight and sixteen hours postsurgery), and all surgeons followed the same deep vein thrombosis prophylaxis guidelines according to patient-specific risk.

### 2.4. Outcomes

Preoperative and postoperative (6- to 12-month) functional outcomes were obtained prospectively. The Oxford hip score (OHS) [13], forgotten joint score (FJS) [14], EuroQoL 5-dimensional (EQ-5D) score [15], EQ visual analogue scale (VAS) [15], level of pain, and patient satisfaction with their hip were assessed. The OHS, FJS, EQ-5D, EQ-VAS, and level of pain were assessed pre- and postoperatively.

The OHS is a hip-specific score and was used as the primary outcome measure. This score consists of twelve questions assessed on a Likert scale with values from 0 to 4, and a summative score is then calculated where 48 is the best possible score (least symptomatic) and 0 is the worst possible score (most symptomatic). [13] The OHS has a defined minimal clinically important difference of 5 points. [16] The FJS assesses joint awareness during the activities of daily living (e.g., climbing stairs and walking for more than 15 minutes, in bed at night). [14] It consists of 12 questions assessed using a five-point Likert response format. Item scores are summed and linearly transformed to a 0 to 100 scale, a high value reflecting the ability of the patient to forget about the affected/replaced joint during the activities of daily living. The EQ-5D 3-level was used, which measures five domains (mobility, self-care, usual activities, pain/discomfort, and anxiety/depression) according to three levels (3L) of severity. [15] An individual patient's health state can be reported based on the five-digit code for each domain, of which there are 243 possible health states (ranging from -0.56, which is worse than death, to 1.0 being perfect health). The EQ-VAS was assessed, which records the patient's self-rated health on a 20 cm vertical visual analogue scale, where the endpoints are labelled “The best health you can imagine” (100) and “The worst health you can imagine” (0). The EQ-VAS can be used as a quantitative measure of health outcome that reflects the patient's own judgement.

### 2.5. QALYs Gained

The EQ-5D 3-level was used as the preference-based measure to assess QALYs gained following THA. The regression analysis was used to identify the independent effect of rTHA on change in the EQ-5D score postoperatively relative to mTHA, which was defined as the QALY gained at one year (uplift). Two models for incremental cost-effectiveness ratios were then created. The first model was constructed over a ten-year period postoperatively to assess the cost per QALY of rTHA relative to mTHA. The QALY gain at one year was then multiplied by the number of years following surgery up to a maximum of 10 and was adjusted for mortality and revision (diminishing patients with time). The mortality rate was defined a 25% at 10 years (2.5% per year) [17]. The data from the NJR 17th Annual Report were used to define a 10-year revision rate of 3.89%, which was calculated assuming a 40% males, with an associated revision rate of 4.28% at 10 years, and a 60% females, with an associated revision rate of 3.63% at 10 years for all THA aged 65 to 74 years [8]. A second model was created to calculate lifetime cost per QALY of rTHA relative to mTHA. The average remaining life expectancy of a 69-year-old patient (the average age of patients undergoing THA in the NJR [8]) was obtained from the Office of National Statistics, which was 16 years for males and 18 years for females, and therefore, the average of 17 years of life expectancy following THA was employed [18]. The annual revision rate was calculated from the average 10-year revision rate used in model one being defined as 0.389 per year. Discounting of 5% per year for quality of health gain was applied to the 10-year model and lifetime models, which are adjusted for diminishing health gain with time [19]. As the cost of the robot was fixed and the per patient cost would be proportional to the number of rTHAs performed, it was assumed that a unit would undertake 100 rTHAs annually for the lifetime cost per QALY model.

### 2.6. Costs

The cost of the robotic equipment and associated consumables was obtained directly from the provider of the rTHA (MAKO Surgical Corporation, Fort Lauderdale, FL). There are two options of purchasing the robot: outright purchase or monthly rental cost (which covers maintenance contract) or a monthly cost of £9,600, which was used in the cost-utility analysis with an annual cost of £115,200 (personal communication with Stryker). The costs were then summed and divided by the defined base case volume of cases per year. Then, the cost of the consumables (£278) was calculated per patient according to case volume (personal communication with Stryker). The cost of a preoperative computed tomographic scan of three regions for the robotic-assisted group was taken from the UK NHS national tariff (£86). [20] The cost of revision THA was obtained from actual costs sustained by the NHS using data from Vanhegan et al. [21] that demonstrated aseptic cases cost £11,897 and septic cases cost £21,937. Using data from the NJR, it was assumed that 13% of the revisions were septic and 87% were for aseptic reasons [8].

### 2.7. Statistical Analysis

Data analysis was performed using Statistical Package for Social Sciences version 17.0 (SPSS Inc., Chicago, IL, USA). The parametric and nonparametric tests were used as appropriate to assess continuous variables for significant differences between groups. Student's t-test, unpaired and paired, was used to compare linear variables between groups. The dichotomous variables were assessed using a chi-square test. The linear regression analysis was used to adjust for confounding variables influencing change in the EQ-5D to assess the independent effect of rTHA. A *p* value of <0.05 was defined as significant.

## 3. Results

Patients undergoing rTHA were more likely to be male, younger in age, and had greater (better) preoperative EQ-5D and EQ-VAS scores when compared to those undergoing mTHA (Table 1). However, there were no differences in the joint-specific (OHS and FJS) scores between the two groups (Table 1). The rTHA group had significantly better mean postoperative EQ-5D utility compared with the mTHA group, but the overall unadjusted change/improvement was not significantly different between the groups (Table 2). The factors associated with change in the EQ-5D following surgery were the preoperative OHS, FJS, EQ-5D, and EQ-VAS scores (Table 3), with greater (better) scores being related to a smaller change in the EQ-5D utility. When adjusting for confounding variables (sex, age, preoperative patient-reported outcome measures), rTHA was associated with a 0.091-point greater improvement in the EQ-5D postoperative when compared to mTHA (Table 4). This resulted in a cost per QALY for rTHA using the base model (10-year follow-up in a unit performing 100 rTHAs per year) of £1,910 undiscounted and £2,349 when including a 5% per year discount of QALY gained relative to mTHA (Table 5).

**Figure 1:**
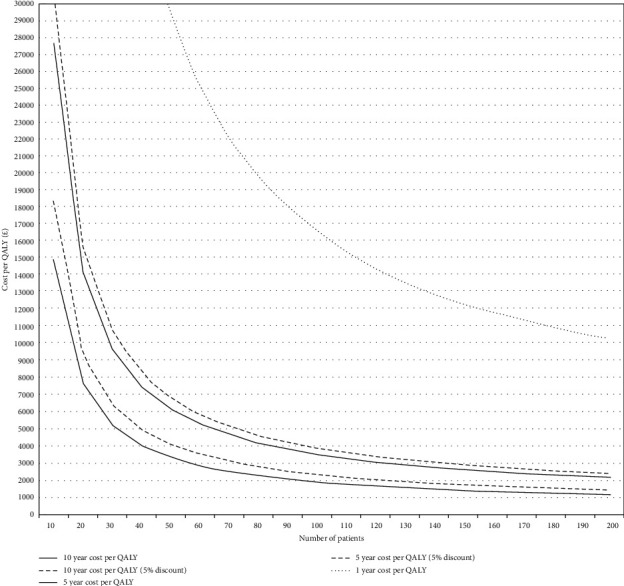
Cost per QALY for robotic relative to manual total hip arthroplasty according to annual unit volume and the time point used to define QALYs gained following surgery.

The cost per QALY of rTHA relative to mTHA was influenced by the number of patients per year a centre undertakes (due to the fixed cost of robot per year) and the defined follow-up period (Figure 1). When using the 10-year time point for QALYs gained, a centre undertaking 10 or more rTHAs remained under the £20,000 cost per QALY (both undiscounted and discounted), with a cost per QALY of approximately £3,000 for centres undertaking 50 rTHAs and £1,000 for units undertaking 200 rTHAs per year.

The increased cost per patient for rTHA relative to mTHA for a centre undertaking 100 procedures per year was £1,516 (Table 5). There was a 0.370 improvement in the EQ-5D score following mTHA, which was used to calculate the QALYs gained over the remaining life expectancy of a 69-year-old patient when accounting for revision costs and a 5% annual discount (deterioration of improved health gain with time) (Table 5). The uplift in the EQ-5D (Table 5) associated with rTHA (0.091) was used to calculate the QALYs gained. The lifetime cost per QALY for rTHA was £980 unadjusted and £1,432 adjusted (5% disutility) compared with mTHA (Table 5).

## 4. Discussion

This study has demonstrated that rTHA was a cost-effective intervention. The cost per QALY for rTHA relative to mTHA was between £1,910 and £2,349 at 10 years following surgery for a centre performing 100 per year; however, this was shown to vary according to the length of follow-up and number performed per year. Despite the increased cost associated with rTHA, the lifetime cost per QALY for rTHA was £980, or £1,432 when discounted, compared with mTHA, which was driven by the relative increased health-related quality-of-life gain associated with rTHA.

The non-randomisation of the patients to either rTHA or mTHA is major limitation of this study and was dependent on whether they could financially afford private health care at one hospital or whether they used the NHS in the UK, respectively. However, to conduct a randomised control trial powered to the EQ-5D with a known minimal clinical important difference (MCID) of 0.08 [22] and standard deviation of 0.3, it would require a cohort of 444 patients (222 in each group) to achieve a power of 80% with an alpha of 0.05. This number would increase when adjusting for loss to follow-up. The multivariable regression analysis was used to adjust for preoperative demographics, hip-specific function, and general health, which is a novel aspect of this study relative to other studies comparing rTHA with mTHA using a semi-active system. [11, 12] The relatively short length of follow-up, at a maximum of 12 months, is another limitation, and with longer follow-up, the general quality of health score may continue to improve; however, this may be marginal with no change being observed from 12 to 96 months after mTHA. [23] Furthermore, there will likely be a decline in the benefit observed after the THA, but this was accounted for in this study with a 5% per year discount in QALYs gained. The involvement of other joints such as knee osteoarthritis or contralateral hip was not considered, and if present, these may have influenced the patients' health-related quality of life; however, it may be expected that the rate of “other” joint arthritis may have been the same in both groups.

The health-related quality-of-life gain of 0.091 in the EQ-5D index associated with rTHA relative to mTHA was greater than the MCID for the score. [22] This increased health gain resulted in greater QALYs gained in the rTHA group, which subsequently resulted in a lower cost per QALY over a typical patient's lifetime despite the increased cost. The gain of 0.091 was higher than that observed in a previous study by Clement et al. [4] who demonstrated a 0.017 difference between rTHA and mTHA. Studies by Bukowski et al. [12] and Domb et al. [11] compared postoperative SF-12 scores between rTHA and mTHA and converting these to an EQ-5D score using a validated formula by Sullivan et al. [24] demonstrated a greater improvement in the EQ-5D score of 0.024 and 0.045, respectively, in those undergoing rTHA. Therefore, the health gain in this study was more than twice that observed previously. This may be due to the higher preoperative EQ-5D scores observed in those undergoing rTHA who therefore have less of a range to improve postoperatively [4], which was adjusted for in this study. This higher preoperative score is likely due to the younger age [4] and fewer comorbidities, as those undergoing rTHA were approximately 10 years younger than the average age of 69 years. [8] This study adjusted for age and preoperative EQ-5D (a marker of comorbidity [25]) in the regression modelling and rTHA was associated with 0.091 greater improvement postoperatively. Nonetheless, even using the lower improvement of 0.017 in the EQ-5D demonstrated by Clement et al. [4] the lifetime cost per QALY of rTHA would be £5246 compared with £980 when employing the same model used in this study.

The lifetime cost per QALY of mTHA in the NHS in the UK is estimated to be £1,372 and £3,763 when discounted by 5%, which was calculated using the procedural cost of £8,956 from the Scottish National Tariff. [7] The NHS England tariff cost of mTHA is less than that in Scotland where best price tariff ranges from £5,870 to £6,307 depending on associated comorbidity; therefore, the cost per QALY may be less than that suggested using the Scottish tariff. Nonetheless, adding the identified incremental cost per QALY for rTHA to the established cost for mTHA the estimated cost per QALY would be £2,352 and £5,195 when discounted by 5% for rTHA. Therefore, the cost per QALY of rTHA is far below the £20,000 upper limit set by the National Institute of Clinical Excellence. [26] To put this into context further, rTHA had an incremental cost per QALY similar to cataract surgery [27], which is regarded as one of the most cost-effective interventions in medicine. [28]

To the authors' knowledge, the only other cost-effective study for rTHA is by Maldonado et al. [6]; they constructed a Markov model to calculate the cost per QALY over a 5-year postoperative period relative to mTHA in an American healthcare system. They found rTHA to be cost-effective relative to mTHA; however, the cost of the robot was not accounted for in their modelling. This study has shown a correlation between the number of rTHAs undertaken in a centre and the cost of the robot, with increasing number of procedures resulting in lower overall cost (the cost of the robot is shared among the patient group). However, even centres performing 10 rTHAs per year were shown to be cost-effective and had a cost per QALY of less than £20,000 at 10-year follow-up. Conversely, the lifetime cost per QALY model was for a centre performing 100 rTHAs per year, and therefore, the cost per QALY of £980 and £1,432 discounted costs presented in this study would be less for units performing more than 100 per year.

This study assumed an equal complication and revision rates for both rTHA and mTHA, which may not be the case. A recent meta-analysis identified a trend towards a lower rate of dislocation (0.4% versus 1.4%) and revision rate associated with rTHA compared with mTHA. [5] Potentially, if this were the case this would decrease the cost burden in the rTHA group and decrease the cost per QALY, making it more cost-effective. Burn et al. [29] assessed the influence of a 50% reduction in revision rate and found a threshold price of £1,347 per patient, therefore provided the increased cost associated with rTHA was below this it would be considered cost-effective. Furthermore, the same group also assessed the influence of a 5% improvement in health-related quality of life, which demonstrated a threshold value of £10,578 below which rTHA would be a cost-effective intervention. [29] This study demonstrated a 25% (0.091/0.370) greater improvement in quality of life associated with rTHA compared with mTHA. This was beyond the 5% modelled by Burn et al. [29] supporting the assumption that rTHA is a cost-effective intervention, as the increased cost per patient of rTHA in this study for a unit performing 100 per year was £1,516 and was below their suggested threshold value of £10,578.

## 5. Conclusion

Despite the increased cost associated with rTHA, it was a cost-effective intervention relative to mTHA at both 10-year follow-up and for a lifetime horizon and was under the threshold of £20,000 per QALY gained. However, the results of this study should be affirmed in larger studies such as a randomised controlled trial before widespread adoption of rTHA is established in the NHS.

## Figures and Tables

**Table 1:** Patient demographics and preoperative functional scores according to group.

Demographic	Descriptive		Group		Odds ratio/difference (95% CI)	*p* value
			mTHA (*n* = 512)	rTHA (*n* = 48)		
Sex (*n*, % of group)		Male	235 (45.9)	32 (66.7)	Reference	
		Female	277 (54.1)	16 (33.3)	OR 0.43 (0.23 to 0.79)	0.006^*∗*^

Age (years: mean, SD)			67.5 (12.1)	58.9 (7.9)	Diff 8.6 (4.8 to 12.4)	<0.001^*∗∗*^

Preoperative PROMs (mean, SD)						
OHS			20.9 (8.7)	19.6 (14.3)	Diff 1.3 (−1.7 to 4.3)	0.393^*∗∗*^
FJS			12.3 (15.5)	12.1 (11.3)	Diff 0.2 (−4.8 to 5.1)	0.947^*∗∗*^
EQ-5D			0.384 (0.312)	0.630 (0.196)	Diff 0.245 (0.14 to 0.346)	<0.001^*∗∗*^
EQ-VAS			68.3 (22.1)	77.5 (13.9)	Diff 9.2 (2.2 to 16.3)	0.011^*∗∗*^

^*∗*^Chi-square test, ^*∗∗*^unpaired *t*-test, PROMs: patient-reported outcome measures.

**Table 2:** Mean pre- and postoperative EQ-5D utility scores according to group. The change represents the improvement between the pre- and postoperative EQ-5D utilities, and the difference represents the difference between the groups pre- and postoperative and for change in the EQ-5D utilities.

Group	Time of assessment (mean, SD)		Change (95% CI)	*p* value
	Preoperative	Postoperative		

mTHA	0.384 (0.320)	0.754 (0.263)	0.370 (0.340 to 0.400)	<0.001^*∗*^
rTHA	0.630 (0.196)	0.905 (0.139)	0.275 (0.215 to 0.336)	<0.001^*∗*^
Difference (95% CI)	0.245 (0.144 to 0.346)	0.148 (0.144 to 0.346)	0.095 (−0.011 to 0.200)	
*p* value	<0.001^*∗∗*^	<0.001^*∗∗*^	0.078^*∗∗*^	

^*∗*^Paired *t*-test, ^*∗∗*^unpaired *t*-test, SD: standard deviation, CI: confidence interval.

**Table 3:** Change in the EQ-5D score according to patient demographics, preoperative functional scores, and type of total hip arthroplasty (robotic arm-assisted versus non-robotic arm-assisted).

Demographic		Descriptive	Correlation/difference (95% CI)	*p* value
Sex (*n*, % of group)		Male	Reference	
		Female	Diff 0.045 (−0.012 to 0.101)	0.121^*∗*^

Age			Corr 0.007 (−0.077 to 0.090)	0.873^*∗∗*^

Preoperative PROMs				
OHS		Corr −0.345 (−0.416 to −0.269)	<0.001^*∗∗*^
FJS		Corr −0.247 (−0.324 to −0.167)	<0.001^*∗∗*^
EQ-5D		Corr −0.679 (−0.722 to −0.631)	<0.001^*∗∗*^
EQ-VAS		Corr −0.155 (−0.235 to 0.072)	<0.001^*∗∗*^

rTHA (*n*, % of group)		No	Reference	
		Yes	Diff −0.095 (−0.200 to 0.11)	0.078^*∗*^

^*∗*^Chi-square test, ^*∗∗*^Pearson's correlation, PROMs: patient-reported outcome measures.

**Table 4:** Linear regression analysis of patient demographics, preoperative functional scores, and type of total hip arthroplasty (robotic arm-assisted versus non-robotic arm-assisted) associated with change in the EQ-5D score postoperatively.

Demographic	Descriptive	Change in EQ-5D (95% CI)	*p* value
Sex (*n*, % of group)	Male	Reference	
	Female	0.005 (−0.037 to 0.047)	0.814
Age	0.000 (−0.002 to 0.001)	0.540
Preoperative PROMs			
	OHS	0.005 (0.002 to 0.009)	0.005
	FJS	0.000 (−0.003 to 0.001)	0.313
	EQ-5D	−0.829 (−0.916 to −0.743)	<0.001
	EQ-VAS	0.002 (0.001 to 0.003)	0.004
rTHA (*n*, % of group)	No	Reference	
	Yes	0.091 (0.009 to 0.173)	0.029

PROMs: patient-reported outcome measures.

**Table 5:** Health economic analysis for lifetime cost per QALY for manual total hip arthroplasty (mTHA) and robotic arm-assisted total hip arthroplasty (rTHA).

Health economic analysis	
*Utility*	
Mean (SD) health evaluation	
mTHA preoperative	0.384 (0.320)
mTHA postoperative	0.754 (0.263)
mTHA mean difference (95% CI)	0.370 (0.340 to 0.400)
	*p* < 0.001^*∗*^
rTHA uplift (Table 4)	0.091

*Mortality*	
10-year mortality rate	25%
Remaining life expectancy of a 69-year-old patient	17 years

*Financial costs*	
Cost of mTHA (NHS tariff)	£6,207
Additional costs of rTHA	
Robot (based on 100 per year)	£1,152
Consumables	£278
CT scan	£86
Total per patient	£1516

*Rate and cost of revision*	
Annual revision rate	0.389%
Revision costs	
Aseptic (87% cases)	£11,897
Septic (13% cases)	£21,937

*QALYs gained*	
10-year QALY gain ^*∗∗*^	
Undiscounted	0.7935
Discounted (5%)	0.6453
Lifetime QALY gain	
Undiscounted	1.5470
Discounted (5%)	1.0590

*Cost per QALY*	
10-year horizon	
Undiscounted	£1,910
Discounted	£2,349
Lifetime horizon	
Undiscounted	£980
Discounted	£1,432

^*∗*^ Paired *t*-test, ^*∗∗*^ accounts for revision rate and mortality.

## Data Availability

Data are not publicly accessible in view of patient confidentiality.

## References

[1] Learmonth I. D., Young C., Rorabeck C. (2007). The operation of the century: total hip replacement. *The Lancet*.

[2] Perets I., Mu B. H., Mont M. A., Rivkin G., Kandel L., Domb B. G. (2020). Current topics in robotic-assisted total hip arthroplasty: a review. *Hip International*.

[3] Kouyoumdjian P., Mansour J., Assi C., Caton J., Lustig S., Coulomb R. (2020). Current concepts in robotic total hip arthroplasty. *SICOT J*.

[4] Clement N. D., Gaston P., Bell A. (2021). Robotic arm-assisted versus manual total hip arthroplasty. *Bone Joint Res*.

[5] Ng N., Clement N. D., Gaston P., Simpson P., Macpherson G. J., Patton J. T. (2021). Robotic-arm assisted versus manual total hip arthroplasty: a systematic review and meta-analysis. *Bone Joint J*.

[6] Maldonado D. R., Go C. C., Kyin C. (2020). Robotic arm-assisted total hip arthroplasty is more cost-effective than manual total hip arthroplasty: a Markov model analysis. *Journal of the American Academy of Orthopaedic Surgeons*.

[7] Jenkins P. J., Clement N. D., Hamilton D. F., Gaston P., Patton J. T., Howie C. R. (2013). Predicting the cost-effectiveness of total hip and knee replacement: a health economic analysis. *Bone Joint J*.

[8] Ben-Shlomo Y., Blom A., Boulton C. (2019). *“The National Joint Registry 16th Annual Report 2019”*.

[9] Dimitriou D., Antoniadis A., Flury A., Liebhauser M., Helmy N. (2018). Total hip arthroplasty improves the quality-adjusted life years in patients who exceeded the estimated life expectancy. *The Journal of Arthroplasty*.

[10] Sassi F. (2006). Calculating QALYs, comparing QALY and DALY calculations. *Health Policy and Planning*.

[11] Domb B. G., Chen J. W., Lall A. C., Perets I., Maldonado D. R. (2020). Minimum 5-year outcomes of robotic-assisted primary total hip arthroplasty with a nested comparison against manual primary total hip arthroplasty: a propensity score-matched study. *Journal of the American Academy of Orthopaedic Surgeons*.

[12] Bukowski B. R., Anderson P., Khlopas A., Chughtai M., Mont M. A., Illgen R. L. (2016). Improved functional outcomes with robotic compared with manual total hip arthroplasty. *Surgical Technology International*.

[13] Dawson J., Fitzpatrick R., Carr A., Murray D. (1996). Questionnaire on the perceptions of patients about total hip replacement. *J Bone Joint Surg Br*.

[14] Behrend H., Giesinger K., Giesinger J. M., Kuster M. S. (2012). The “forgotten joint” as the ultimate goal in joint arthroplasty: validation of a new patient-reported outcome measure. *The Journal of Arthroplasty*.

[15] Brooks R. (1996). EuroQol: The current state of play. *Health Policy*.

[16] Beard D. J., Harris K., Dawson J. (2015). Meaningful changes for the Oxford hip and knee scores after joint replacement surgery. *Journal of Clinical Epidemiology*.

[17] Ramiah R. D., Ashmore A. M., Whitley E., Bannister G. C. (2007). Ten-year life expectancy after primary total hip replacement. *J Bone Joint Surg Br*.

[18] Office for National Statistics (2021). Living Longer is age 70 the new age 65. https://www.ons.gov.uk/peoplepopulationandcommunity/birthsdeathsandmarriages/ageing/articles/livinglongerisage70thenewage65/2019-11-19.

[19] Attema A. E., Brouwer W. B. F., Claxton K. (2018). Discounting in economic evaluations. *PharmacoEconomics*.

[20] Office for National Statistics (2018). *“Home People, Population and Community Births, Deaths and Marriages Life Expectancies National Life Tables, UK National Life Tables, UK: 2014 to 2016 Trends in the Average Number of Years People will live beyond their Current Age Measured by Period life Expectancy, Analysed by Age and Sex for the UK and its Constituent Countries,”*.

[21] Vanhegan I. S., Malik A. K., Jayakumar P., Ul I. S., Haddad F. S. (2012). A financial analysis of revision hip arthroplasty: the economic burden in relation to the national tariff. *J Bone Joint Surg Br*.

[22] Larsen K., Hansen T. B., Soballe K. (2008). Hip arthroplasty patients benefit from accelerated perioperative care and rehabilitation: a quasi-experimental study of 98 patients. *Acta Orthopaedica*.

[23] Field R. E., Cronin M. D., Singh P. J. (2005). The Oxford hip scores for primary and revision hip replacement. *J Bone Joint Surg Br*.

[24] Sullivan P. W., Ghushchyan V. (2006). Mapping the EQ-5D index from the SF-12: US general population preferences in a nationally representative sample. *Medical Decision Making*.

[25] Hays R. D., Bjorner J. B., Revicki D. A., Spritzer K. L., Cella D. (2009). Development of physical and mental health summary scores from the patient-reported outcomes measurement information system (PROMIS) global items. *Quality of Life Research*.

[26] McCabe C., Claxton K., Culyer A. J. (2008). The NICE cost-effectiveness threshold: what it is and what that means. *PharmacoEconomics*.

[27] Rasanen P., Krootila K., Sintonen H. (2006). Cost-utility of routine cataract surgery. *Health and Quality of Life Outcomes*.

[28] Prinja S., Nandi A., Horton S., Levin C., Laxminarayan R. (2015). *Costs, Effectiveness, and Cost-Effectiveness of Selected Surgical Procedures and Platforms*.

[29] Burn E., Prieto-Alhambra D., Hamilton T. W., Kennedy J. A., Murray D. W., Pinedo-Villanueva R. (2020). Threshold for computer-and robot-assisted knee and hip replacements in the English national health service. *Value in Health*.

